# TARBP2 Suppresses Ubiquitin-Proteasomal Degradation of HIF-1α in Breast Cancer

**DOI:** 10.3390/ijms23010208

**Published:** 2021-12-24

**Authors:** Jie-Ning Li, Pai-Sheng Chen, Ching-Feng Chiu, Yu-Jhen Lyu, Chiao Lo, Li-Wei Tsai, Ming-Yang Wang

**Affiliations:** 1 Institute of Basic Medical Sciences, College of Medicine, National Cheng Kung University, Tainan 70101, Taiwan; jessyli61621@gmail.com (J.-N.L.); bio.benson@gmail.com (P.-S.C.); 2 Department of Medical Laboratory Science and Biotechnology, College of Medicine, National Cheng Kung University, Tainan 70101, Taiwan; sallyliu96@hotmail.com; 3 Graduate Institute of Metabolism and Obesity Sciences, College of Nutrition, Taipei Medical University, Taipei 11031, Taiwan; chiucf@tmu.edu.tw; 4 Department of Surgery, National Taiwan University Hospital, Taipei 100, Taiwan; dtsurgz6@gmail.com (C.L.); b91401028@gmail.com (L.-W.T.); 5 Department of Surgical Oncology, National Taiwan University Cancer Center, Taipei 100, Taiwan

**Keywords:** breast cancer, HIF-1α, TARBP2, ubiquitinated degradation

## Abstract

TAR (HIV-1) RNA binding protein 2 (TARBP2) is an RNA-binding protein participating in cytoplasmic microRNA processing. Emerging evidence has shown the oncogenic role of TARBP2 in promoting cancer progression, making it an unfavorable prognosis marker for breast cancer. Hypoxia is a hallmark of the tumor microenvironment which induces hypoxia-inducible factor-1α (HIF-1α) for transcriptional regulation. HIF-1α is prone to be rapidly destabilized by the ubiquitination–proteasomal degradation system. In this study, we found that TARBP2 expression is significantly correlated with induced hypoxia signatures in human breast cancer tissues. At a cellular level, HIF-1α protein level was maintained by TARBP2 under either normoxia or hypoxia. Mechanistically, TARBP2 enhanced HIF-1α protein stability through preventing its proteasomal degradation. In addition, downregulation of multiple E3 ligases targeting HIF-1α (VHL, FBXW7, TRAF6) and reduced ubiquitination of HIF-1α were also induced by TARBP2. In support of our clinical findings that TARBP2 is correlated with tumor hypoxia, our IHC staining showed the positive correlation between HIF-1α and TARBP2 in human breast cancer tissues. Taken together, this study indicates the regulatory role of TARBP2 in the ubiquitination–proteasomal degradation of HIF-1α protein in breast cancer.

## 1. Introduction

Hypoxia-inducible factor-1α (HIF-1α) is a versatile transcriptional factor that exerts its transactivation ability for transcriptional regulation [1]. Utilizing its transcriptional activity, HIF-1α affects several cancer properties, including tumorigenesis, apoptosis, genomic instability, metastasis, and invasion, and is therefore important in cancer progression [2]. Involved in numerous cellular processes, HIF-1α is tightly regulated by oxygen concentration. Under normoxic conditions, HIF-1α acts as an oxygen-labile protein with a short half-life, which means that as soon as HIF-1α is synthesized it gets degraded via the proteasomal or lysosomal degradation pathways [3]. HIF-1α is hydroxylated by prolyl-4-hydroxylases (PHDs) in the oxygen-dependent degradation domain (ODDD) at the proline residues (P402 and P564), which is recognized by its E3 ligase, von Hippel–Lindau protein (VHL), followed by ubiquitination, thereby leading to its proteasomal degradation. During hypoxic conditions, PHDs are inhibited and HIF-1α escapes the fate of degradation and becomes dominant to exert its effect. In addition to hypoxia, deubiquitination of HIF-1α is an alternate pathway which inhibits degradation, and this process is facilitated by a group of deubiquitinating enzymes (DUBs). These deubiquitinases (DUBs) function via removing the ubiquitinated marks or editing the ubiquitin chains in the target proteins to recycle ubiquitin for maintaining ubiquitin homeostasis [4]. Consequently, protein degradation controls the main regulation of HIF-1α, which results from the fluctuations in ubiquitination and deubiquitination. Although several E3 ligases and DUBs have been individually reported to affect HIF-1α expression [4], the global regulation of these two processes is less well understood. TAR (HIV-1) RNA binding protein 2 (TARBP2) is a multifunctional RNA-binding protein known to be involved in microRNA (miRNA) biogenesis, viral infection, and tumorigenesis [5]. TARBP2 is one of the miRNA biogenesis factors, participating in the cytoplasmic processing of miRNA and acting as a cofactor with Dicer [6]. TARBP2 also regulates HIV-1 gene expression through interacting with TAR [6]. Goodarzi et al. reported that TARBP2 is overexpressed in metastatic tumor cells and promotes colony formation and invasion ability through destabilizing two metastasis suppressors, including amyloid precursor protein (APP) and ZNF395 [7]. In addition, TARBP2 is reported to promote tumor-induced angiogenesis through degradation of mRNAs coding for antiangiogenetic factors, including thrombospondin1/2 (THBS1/2), tissue inhibitor of metalloproteinases 1 (TIMP1), and serpin family F member 1 (SERPINF1) [8]. TARBP2 has been shown to enhance a transformed phenotype and tumorigenesis in vivo and is considered an unfavorable prognostic marker for breast cancer [9,10]. These studies suggest an oncogenic role of TARBP2 in cancer, which might be involved in numerous unknown mechanisms. Although HIF-1α and TARBP2 are both crucial for cancer development, the relationship between these two proteins remains to be clarified. In this study, we clarify the relationship between TARBP2 and HIF-1α and show that TARBP2 positively regulates HIF-1α by inhibiting its poly-ubiquitination and proteasomal degradation.

## 2. Materials and Methods

### 2.1. Cell Culture

Breast cancer cell line MCF-7 was incubated in Dulbecco’s modified Eagle medium (HiMedia DMEM) with low glucose supplemented with 10% fetal bovine serum (FBS, Corning, New York, NY, USA) and 1% penicillin/streptomycin antibiotics (HyClone). Breast cancer cell line MDA-MB-231 cells were maintained in Dulbecco’s modified Eagle medium (HiMedia DMEM, high glucose) supplemented with 10% fetal bovine serum (FBS, Corning) and 1% penicillin/streptomycin antibiotics (HyClone). All cells were incubated at 37 °C with 5% CO_2_ in a humidified incubator. The MCF-7 and MDA-MB-231 cells were obtained from American Type Culture Collection (ATCC).

### 2.2. Western Blot

After cells reached 70–80% confluence, the whole cell lysates were collected in NETN buffer (20 mM Tris-Base, pH 8.0, 150 mM NaCl, 1 mM EDTA, 0.5% Nonidet P-40) combined with protease inhibitors and phosphatase inhibitors. The proteins were purified by centrifugation at 14,000 rpm, 4 °C and quantified using Dual-Range^TM^ Bradford reagent. Then, 60–70 μg of proteins were fractionated on the 8–12% Tris-glycine gel and electrophoretically transferred onto PVDF membranes according to the manufacture’s protocols (Bio-Rad). After protein transfer, the membranes were blocked with 5% non-fat milk in TBST buffer (20 mM Tris base, 150 mM NaCl, and 0.1% Tween 20) for at least 60 min, and then washed in TBST for 10 min. Membranes were incubated with primary antibodies diluted in 2% BSA in TBST at 4 °C overnight and incubated with anti-mouse or anti-rabbit secondary antibodies for 60 min having been washed with TBST three times for 10 min on the secondary day. Protein expressions were visualized using an ECL detection system according to the manufacture’s protocols. 

### 2.3. RNA Extraction and Reverse Transcriptase Real-Time PCR

Total RNA was extracted by Trizol reagent according to the manufacturer’s instructions and quantified with the NanoDrop Lite spectrophotometer. Complementary DNA (cDNA) was synthesized with 100–200 ng of RNA in an MJ Mini thermal cycler. For reverse transcription, reaction mixtures containing template RNA, random hexamer, and dNTP were incubated at 65 °C for 5 min, following which, reaction buffer, RNase inhibitor, RevertAid RT and nuclease-free water were added for incubation at 25 °C for 5 min and 42 °C for 60 min, terminating at 70 °C for 5 min. The amplifications of cDNA were performed using the SYBR Green PCR Master Mix in an Applied Biosystem Step One Real-time PCR system (Applied Biosystems) according to the manufacturer’s protocols. The reaction mixture, including Fast SYBR™ Green PCR Master Mix, forward primer, reverse primer, nuclease-free water and cDNA templates, was incubated at 95 °C for 20 s, 40 cycles of 95 °C for 3 s and 60 °C for 30 s. Each sample was carried out in duplicate and GAPDH was used to normalize the target genes. 

### 2.4. Tissue Microarray

The tissue array was obtained from Taipei Medical University Joint Biobank and purchased from Biomax US, lnc. (Derwood, MD, USA) Waivers of informed consent were approved by A-ER-106-483 from NCKU Hospital. The study was conducted according to ethical approval by the Ethics Committee of Taipei Medical University (approval numbers N202001052 and N202103065). 

### 2.5. Immunohistochemistry

Breast cancer tissue array was used for immunohistochemistry (IHC) staining. The TARBP2 and HIF-1α antigen retrieval program was incubated in boiling citrate buffer (pH 6.0) for 20 min. Blocking buffer (TA00C2, BioTnA, Kaohsiung, Taiwan) and H_2_O_2_ were used to block endogenous peroxidase of the tissue for 60 min each. Tissue sections were stained with primary antibodies, followed by HRP-conjugated secondary antibodies, to observe TARBP2 (code No. 3-2, customized antibody, Leadgene Biomedical, Tainan, Taiwan) and HIF-1α (BS3514, Bioworld Technology, Inc., St. Louis, MO, USA) at 1:3000 and 1:100 dilution factors. Primary antibodies of TARBP2 and HIF-1α were incubated overnight at 4 ℃ and room temperature, respectively, and expression levels were measured using the TAlink mouse/rabbit polymer detection system (TAHC04D, BioTnA, Kaohsiung, Taiwan). 

### 2.6. Statistical Analysis

All experiments were carried out at least three times and results were calculated as the means ± standard error of mean (SEM). The data was analyzed by *t*-tests for two individual groups or two-way ANOVA for groups with two variables in Prism7 software. Values between different groups were considered significant when *p* was less than 0.05.

## 3. Results

### 3.1. TARBP2 Upregulates HIF-1α Expression

**Figure 1:**
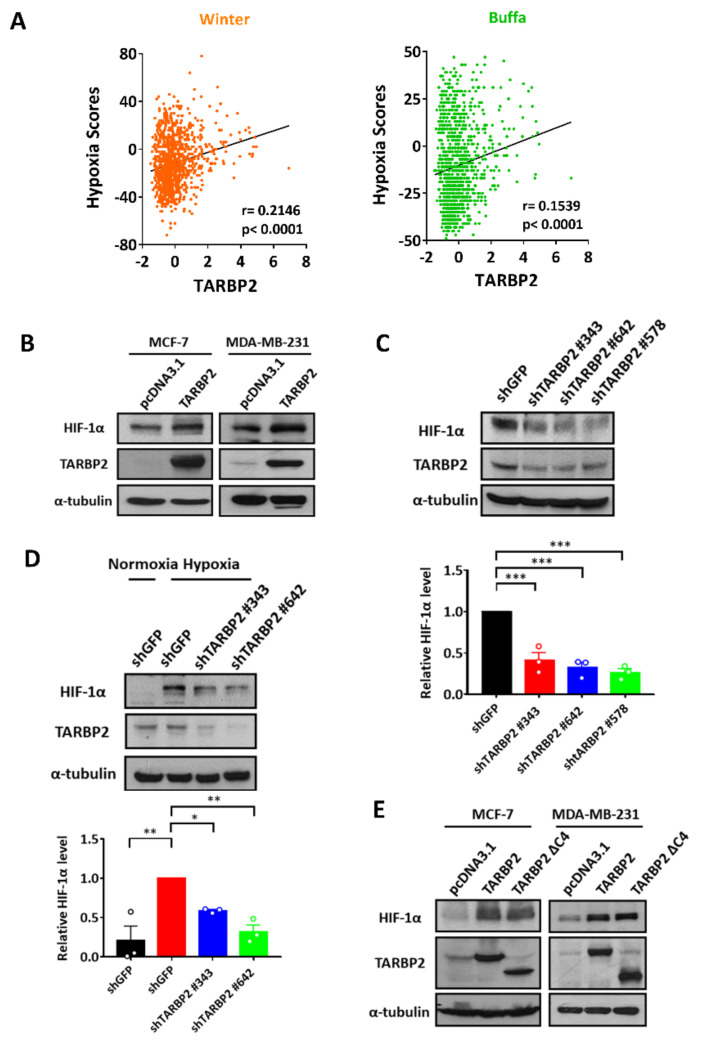
TARBP2 upregulates HIF-1α expression in normoxic and hypoxic conditions. (**A**) Correlation between TARBP2 and hypoxia score using a patient cohort with breast cancer from the TGCA database. Correlation analysis was performed using Pearson’s correlation coefficient. (**B**) TARBP2 induces the expression of HIF-1α. MCF-7 (**left**) and MDA-MB-231 cells (**right**) were transfected with either control or TARBP2 plasmids for 48 h and cells were harvested to determine the expression of TARBP2 and HIF-1α using western blot analysis. (**C**) HIF-1α expression in TARBP2 knockdown MCF-7 cells. MCF-7 cells were applied to indicated shRNAs targeting TARBP2 for 48 h. After selection using puromycin, the efficiency of TARBP2 knockdown and HIF-1α expression was examined using a western blot. The results of the western blot analyses were quantified and presented in the bottom panel. (**D**) Effect of TARBP2 expression on hypoxia-induced HIF-1α. MCF-7 cells were incubated under hypoxic conditions for 6 h, and the expression of TARBP2 and HIF-1α was determined with a western blot analysis. (**E**) Effect of C4-truncated TARBP2 on HIF-1α expression. MCF-7 (**left**) and MDA-MB-231 cells (**right**) were transfected with the indicated plasmids for 48 h and cells were harvested to determine the expression of TARBP2 and HIF-1α with a western blot. * *p* < 0.05, ** *p* < 0.01, *** *p* < 0.001.

To investigate the association between TARBP2 and hypoxia, we utilized the cohort of breast cancer patients from the Cancer Genome Atlas (TCGA) and analyzed hypoxia scores, which were calculated using the mRNA-based signatures developed by Buffa and Winter [11,12]. TARBP2 was found to be positively correlated with both Winter (Pearson correlation coefficient r = 0.2146, *p* < 0.0001) and Buffa (Pearson correlation coefficient r = 0.1539, *p* < 0.0001) hypoxia scores, which indicated that TARBP2 exhibits a crucial role in hypoxia (Figure 1A). Since HIF-1α is a master transcription factor that is dominant in hypoxia [13], we investigated the expression of HIF-1α in TARBP2-overexpressing cells. Increased HIF-1α expression was observed following TARBP2 overexpression in MCF-7 and MDA-MB-231 cells (Figure 1B). Additionally, decreased HIF-1α expression was observed in TARBP2 knockdown MCF-7 cells, suggesting that TARBP2 induces HIF-1α expression (Figure 1C). Moreover, elevated HIF-1α expression in hypoxia was found to be abrogated by knockdown of TARBP2 (Figure 1D). These data indicated that TARBP2 plays an important role in upregulating HIF-1α expression under both normoxia and hypoxia. Since TARBP2 is a key factor in cytoplasmic miRNA biogenesis and the C4 domain is required for Dicer-mediated miRNA processing [14], we used the C4 domain-truncated TARBP2 (TARBP2 ΔC4) to study whether its function in miRNA biogenesis is required for HIF-1α regulation. The results showed that increased HIF-1α expression is still observed in TARBP2 ΔC4-overexpressing cells as TARBP2 full length, indicating that TARBP2 acts through an miRNA biogenesis-independent pathway to upregulate HIF-1α (Figure 1E).

### 3.2. TARBP2 Inhibits Proteasomal Degradation of HIF-1α

**Figure 2:**
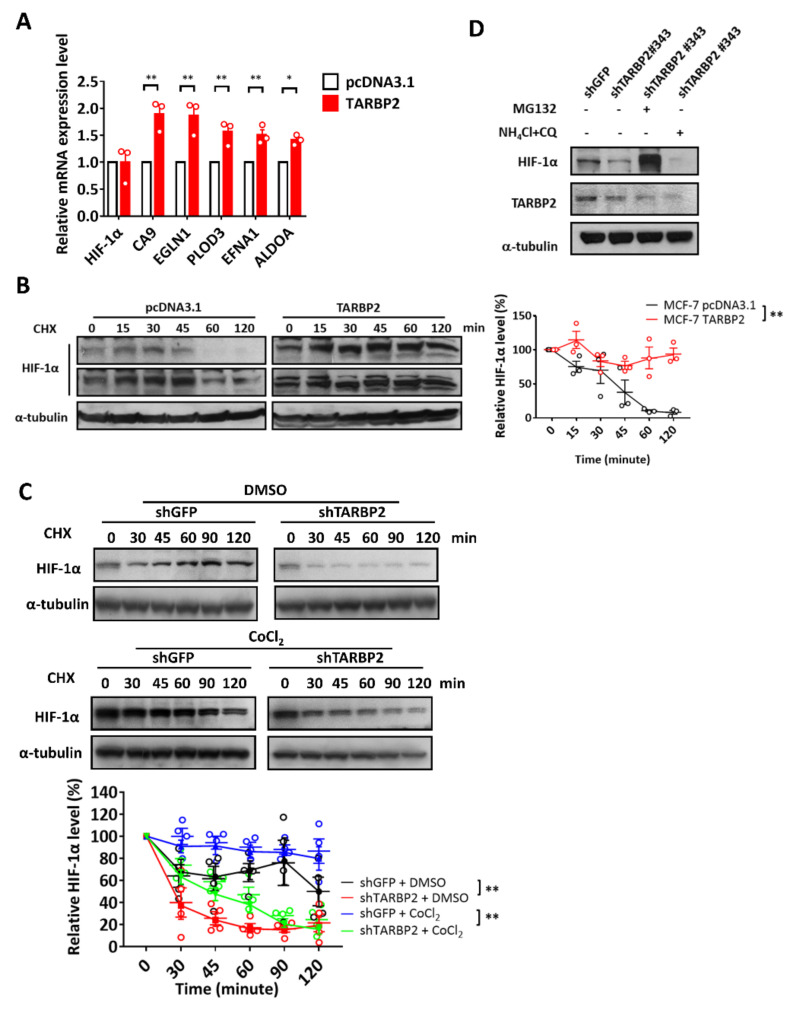
TARBP2 inhibits proteasomal degradation of HIF-1α. (**A**) Relative mRNA expression of HIF-1α and its downstream targets, including CA9, EGLN1, PLOD3, EFNA1 and ALDOA, in TARBP2-overexpressing MCF-7 cells were assessed using qPCR analysis. The stability of HIF-1α protein expression in TARBP2-overexpressing MCF-7 cells (**B**) and CoCl_2_-treated TARBP2-overexpressing MDA-MB-231 cells (**C**,**D**) were investigated upon cycloheximide treatment and harvesting of the cells at the indicated time points. The western blot results from replicate experiments were quantified and analyzed using two-way ANOVA, *p* < 0.01. (**D**) TARBP2 knockdown MCF-7 cells were treated with MG132 or NH_4_Cl/CQ for 24 h to inhibit the proteasomal or lysosomal degradation pathways, respectively. * *p* < 0.05, ** *p* < 0.01.

Since our results showed that TARBP2 promotes HIF-1α protein expression, we investigated whether the mRNA expression of HIF-1α is also affected. Unchanged expression of HIF-1α mRNA was observed in TARBP2-overexpressing cells; however, the downstream expression of HIF-1α, including CA9, EGLN1, PLOD3, EFNA1, and ALDOA, increased (Figure 2A). These results suggest that TARBP2 enhances HIF-1α protein expression as well as its transcriptional activity. To further elucidate the mechanism of TARBP2-mediated upregulation of HIF-1α, we used cycloheximide (CHX) to block its protein synthesis. In TARBP2-overexpressing cells, enhanced protein stability of HIF-1α was observed (Figure 2B). Since the basal level of HIF-1α is relatively low under normoxia, we also treated cells with the hypoxia-mimetic agent CoCl_2_ to enhance HIF-1α accumulation (Figure 2C). In support of our results showing TARBP2-induced HIF-1α under both hypoxia and normoxia, HIF-1α exhibited reduced protein stability when TARBP2 expression was knocked down in both DMSO- and CoCl_2_-treated cells (Figure 2C). HIF-1α is an unstable protein that is rapidly degraded via both proteasomal and lysosomal pathways [13]. Since TARBP2 upregulates HIF-1α through promoting its protein stability, we investigated the underlying pathway using proteasome inhibitor MG132 and lysosome inhibitor NH_4_Cl/ chloroquine (CQ) treatment. In TARBP2 knockdown cells, HIF-1α was found to be downregulated and this phenomenon was abrogated upon MG132 treatment; however, HIF-1α expression remained decreased upon NH_4_Cl/CQ treatment (Figure 2D). These data indicated that TARBP2 upregulates HIF-1α expression through inhibiting its proteasomal degradation, thereby leading to enhanced protein stability and transcriptional activity.

### 3.3. TARBP2 Reduces the Ubiquitination Level of HIF-1α through Downregulating Several E3 Ligases

**Figure 3:**
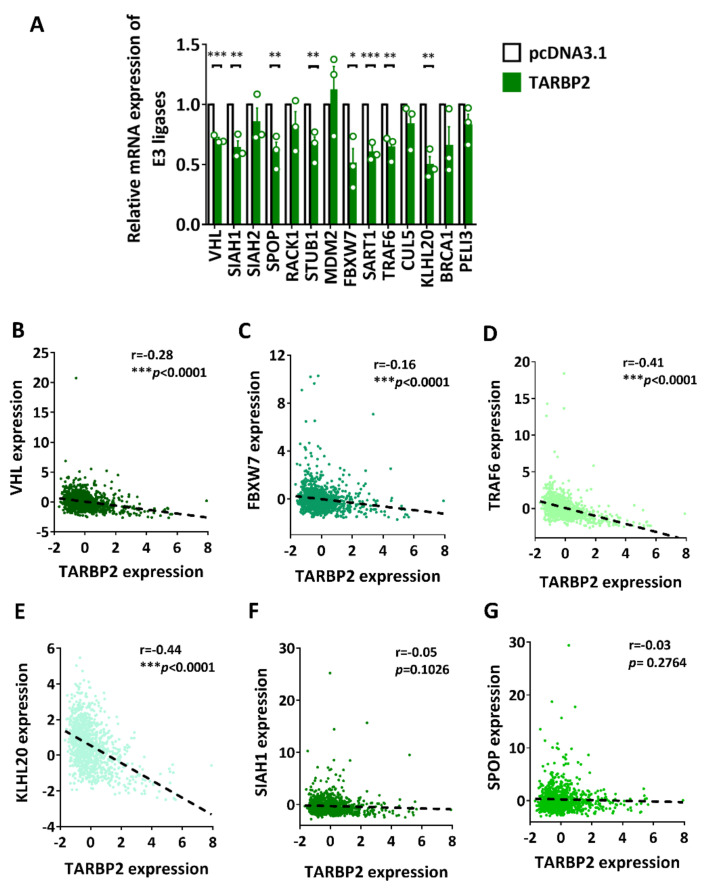

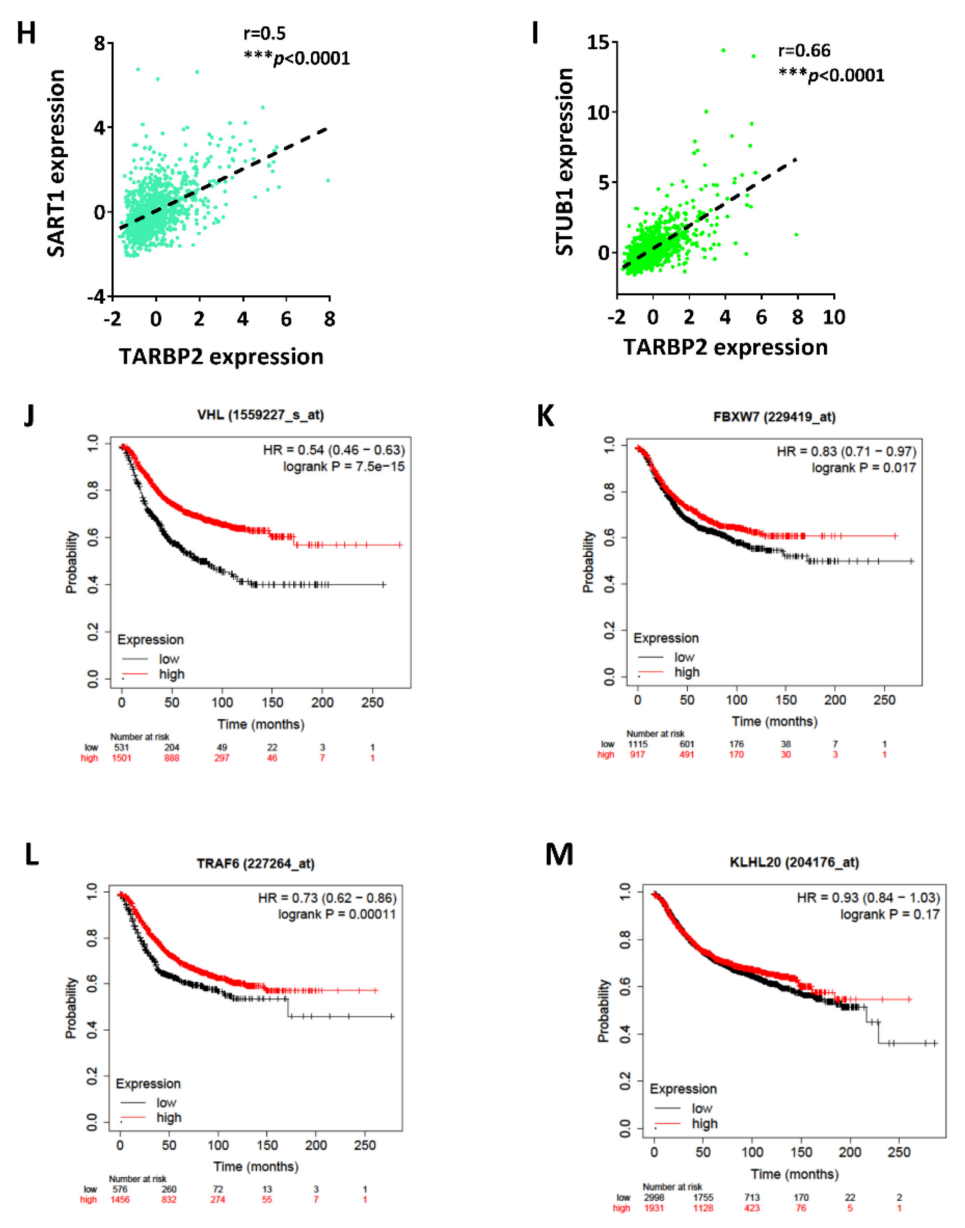
The effect of TARBP2 on the expression of HIF-1α-targeting E3 ligases. (**A**) Relative mRNA expression of HIF-1α-related E3 ligases in TARBP2-overexpressing MCF-7 cells. Correlation between TARBP2 and E3 ligases, including VHL (**B**), FBWX7 (**C**), TRAF6 (**D**), KLHL20 (**E**), SIAH1 (**F**), SPOP (**G**), SART1 (**H**), and STUB1 (**I**), using the cohort of patients with breast cancer from the TCGA database. Kaplan–Meier curves for relapse-free survival (RFS) of patients with VHL-high/low (**J**), FBWX7-high/low (**K**), TRAF6-high/low (**L**), and KLHL20-high/low (**M**) were derived. Correlation analyses were performed using Pearson’s correlation coefficient, * *p* < 0.05, ** *p* < 0.01, *** *p* < 0.001.

During protein degradation, the target protein is ubiquitinated, recognized, and degraded, which suggests that ubiquitination is an essential step in this process [15]. With the assistance of a series of enzymes, ubiquitination marks the specific protein for degradation, and E3 ligases are responsible for target specificity. A group of E3 ligases that are reported to regulate HIF-1α were selected, including VHL, SIAH1, SIAH2, SPOP, RACK1, STUB1, MDM2, FBXW7, SART1, TRAF6, CUL5, KLHL20, BRCA1, and PELI3 [4]. As we discovered that TARBP2 inhibits proteasomal degradation of HIF-1α, we investigated whether these E3 ligases were also affected. First, the mRNA expression of HIF-1α-targeting E3 ligases was evaluated. In TARBP2-overexpressing cells, significant downregulation of several E3 ligases, including VHL, SIAH1, SPOP, STUB1, FBXW7, SART1, TRAF6, and KLHL20, was observed (Figure 3A). These data suggest that TARBP2 may downregulate multiple HIF-1α-related E3 ligases to inhibit the degradation of HIF-1α. Moreover, the correlation between TARBP2 and the significantly downregulated E3 ligases were analyzed using the TCGA breast cancer patient database. Negative correlations between TARBP2 and VHL (r = −0.28, *p* < 0.0001), FBZW7 (r = −0.16, *p* < 0.0001), TRAF6 (r = −0.41, *p* < 0.0001), and KLHL20 (r = −0.44, *p* < 0.0001), which were consistent with in vitro results, were observed in the cohort of human breast cancer tissues (Figure 3B–E), while SIAH1, SPOP, STUB1, and SART1 exhibited no significant correlation with TARBP2 (Figure 3F–I). Additionally, the Kaplan–Meier plotter analysis revealed that VHL (HR = 0.54, *p* < 0.0001), FBZW7 (HR = 0.83, *p* = 0.017), and TRAF6 (HR = 0.73, *p* = 0.0001) were the favorable markers for the prognosis of patients with breast cancer (Figure 3J–L), while KLHL20 makes no contribution to the determination of cancer prognosis (Figure 3M). 

**Figure 4:**
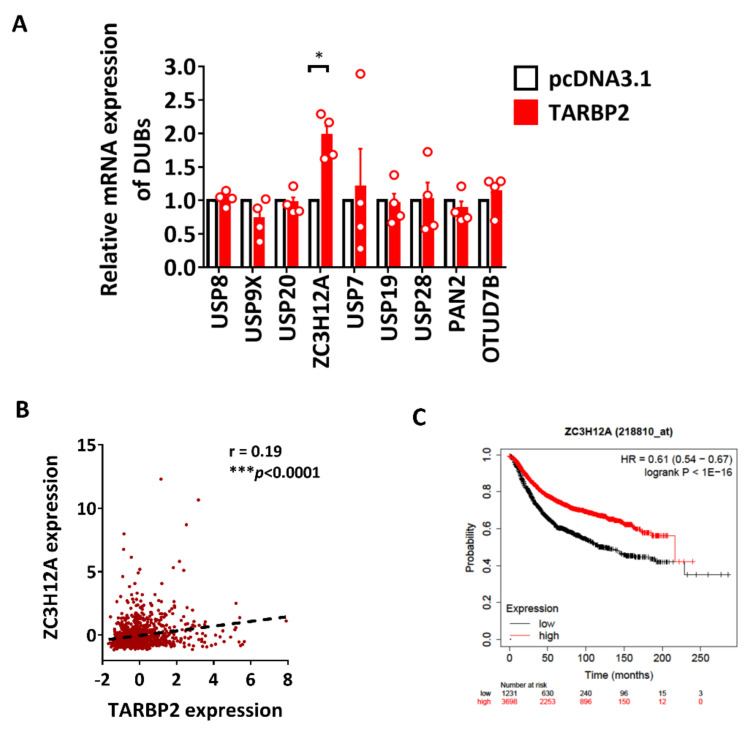
The effect of TARBP2 on the expression of HIF-1α-targeting deubiquitinases. (**A**) Relative mRNA expression of HIF-1α-related deubiquitinases in TARBP2-overexpressing MCF-7 cells. (**B**) Correlation between TARBP2 and ZC3H12A using the cohort of patients with breast cancer from the TCGA database. (**C**) Kaplan–Meier curves for relapse-free survival (RFS) of patients with ZC3H12A-high/low were derived. Correlation analyses were performed using Pearson’s correlation coefficient, * *p* < 0.05, *** *p* < 0.001.

**Figure 5:**
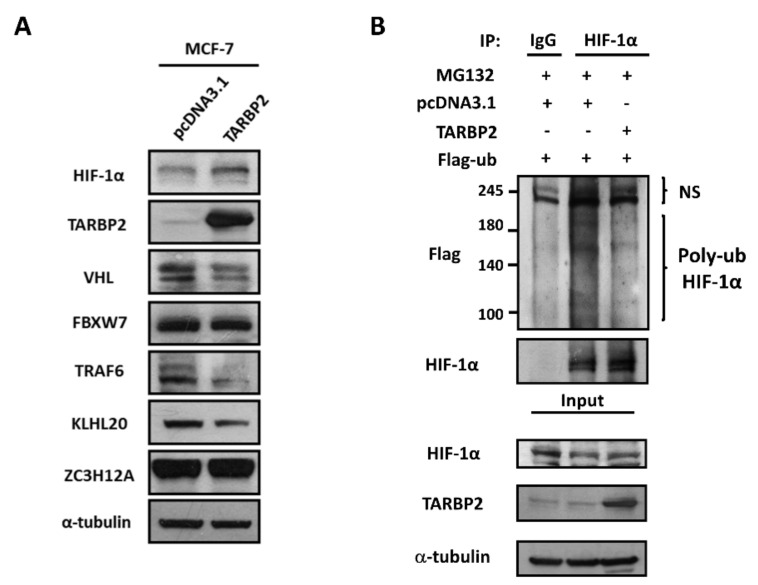
TARBP2 downregulates multiple E3 ligases and HIF-1α ubiquitination. (**A**) Protein expression of HIF-1α, VHL, FBXW7, TRAF6, KLHL20, and ZC3H12A in TARBP2-overexpressing MCF-7 cells. (**B**) Ubiquitination of HIF-1α in TARBP2-overexpressing MCF-7 cells. TARBP2-overexpressing MCF-7 cells were treated with MG132 for 24 h and subjected to immunoprecipitation.

Conversely, the ubiquitin marks on the target can be removed by the deubiquitinases (DUBs), which maintain the homeostasis of the ubiquitin pool [4]. Therefore, USP8, USP9X, USP20, ZC3H12A, USP7, USP19, USP28, PAN2, and OTUD7B were selected as a group of HIF-1α-targeting DUBs for further validation in TARBP2-overexpressing cells. ZC3H12A was significantly upregulated upon TARBP2 overexpression, suggesting the possibility that TARBP2 facilitates deubiquitination of HIF-1α through promoting ZC3H12A expression, which is a deubiquitinase of HIF-1α (Figure 4A). A positive correlation between TARBP2 and ZC3H12A (r = −0.19, *p* < 0.0001) was also observed in human breast cancer tissues (Figure 4B). However, the Kaplan–Meier (KM) plot analysis revealed that ZC3H12A is a favorable marker for the prognosis of patients with breast cancer (Figure 4C), which may counteract the mechanism of TARBP2-mediated deubiquitination of HIF-1α. Collectively, we select VHL, FBXW7, TRAF6, KLHL20, and ZC3H12A as the candidates for protein expression validation in cells with ectopic TARBP2 expression (Figure 5A). Among these E3 ligases, the protein expression of VHL, TRAF6, and KLHL20 was found to be downregulated by TARBP2, while FBXW7 and ZC3H12A were unaffected (Figure 5A), suggesting that TARBP2 suppresses multiple E3s and may result in reduced ubiquitination of HIF-1α. Since HIF-1α is polyubiquitinated before degradation, and we identified that TARBP2 upregulates HIF-1α through inhibiting its proteasomal degradation (Figure 5B), we therefore investigated the ubiquitination status of HIF-1α and consistently found that poly-ubiquitination of HIF-1α was significantly reduced in TARBP2-overexpressing cells. 

### 3.4. TARBP2 Is Positively Correlated with HIF-1α in Breast Cancer Tissues

**Figure 6:**
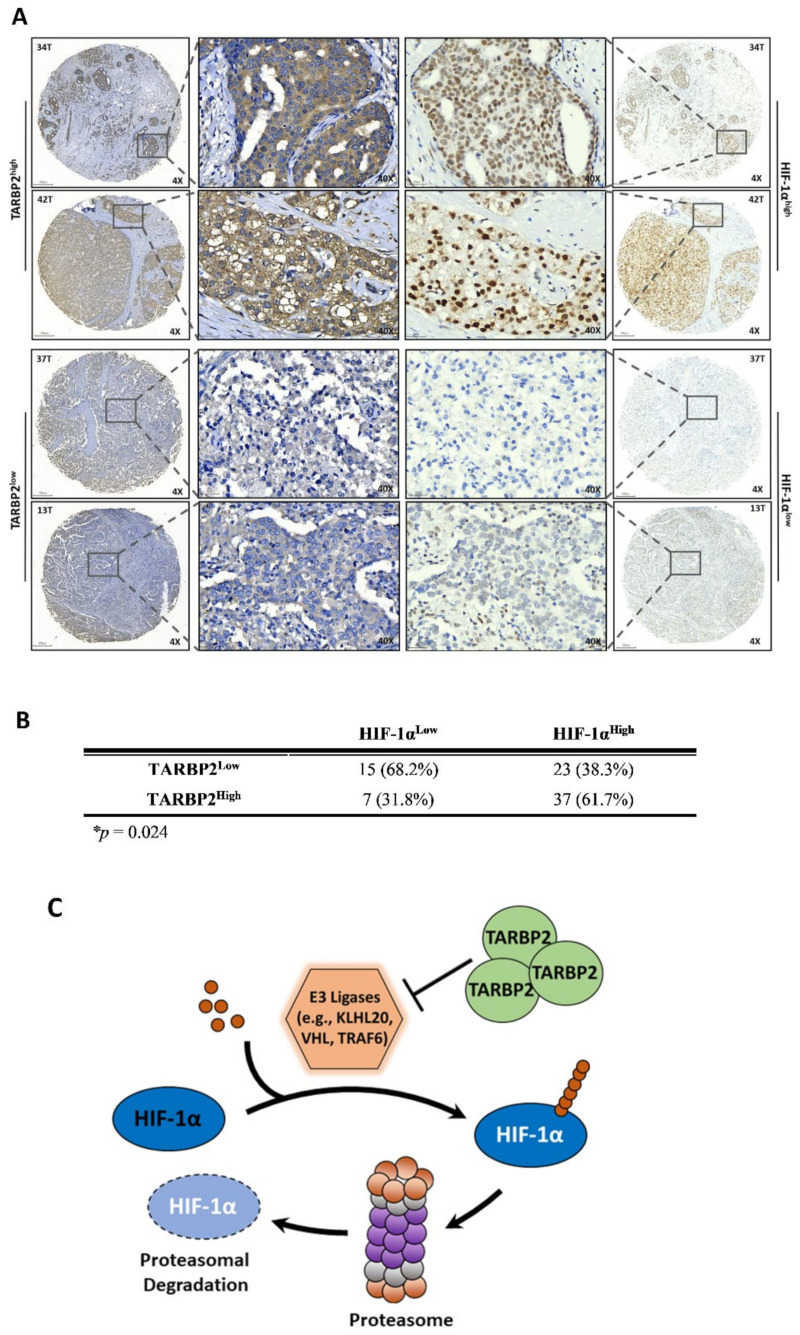
TARBP2 is positively correlated with HIF-1α in human breast cancer. (**A**) Representative images of IHC staining of TARBP2 and HIF-1α in TARBP2^high^ and TARBP2^low^ tumors. The top panel indicates TARBP2^high^ tumors (34T and 42T) and the bottom panel indicates TARBP2^low^ tumors (37T and 13T) with corresponding IHC staining of HIF-1α. (**B**) Quantitated results of IHC images of TARBP2^high^/HIF-1α^high^, TARBP2^high^/HIF-1α^low^, TARBP2^low^/ HIF-1α^high^, and TARBP2^low^/HIF-1α^low^. (**C**) Schematic representation of TARBP2-suppressed HIF-1α degradation.

Having discovered that TARBP2 upregulates HIF-1α through inhibiting its proteasomal degradation, we investigated the correlation between TARBP2 and HIF-1α in patients with breast cancer. The protein expression of TARBP2 and HIF-1α was determined by IHC staining using a tissue microarray of human breast cancer. Higher HIF-1α (HIF-1α^high^) expression was observed in tumor tissues with higher TARBP2 (TARBP2^high^) levels (Figure 6A, upper panel), and vice versa (Figure 6A, bottom panel). The statistical correlation between the IHC scores of TARBP2 and HIF-1α were analyzed (Figure 6B). Compared to the TARBP2^low^ group, the percentage of HIF-1α^high^ was elevated from 38.3% to 61.7% in the TARBP2^high^ group, with decreased HIF-1α^high^ percentage from 68.2% to 31.2% (Figure 6B).

## 4. Conclusions

These data indicated that TARBP2 protein expression is positively correlated with HIF-1α in human breast cancer. Taken together, we revealed that TARBP2 is a HIF-1α positive regulator in both normoxic and hypoxic conditions. Mechanistically, TARBP2 downregulates multiple E3 ligases, reduces HIF-1α ubiquitination and proteasomal degradation of HIF-1α, leading to elevated HIF-1α protein accumulation and transcriptional activation (Figure 6C).

## 5. Discussion

HIF-1α is an important protein that has been shown to play an important role in cancer development. Exhibiting a short half-life, HIF-1α is degraded via the proteasome degradation pathway [16]. The regulation of HIF-1α has been reported previously in several studies focusing on its E3 ligases and deubiquitinases responsible for promoting or inhibiting the degradation pathway [4]. VHL, a well-studied E3 ligase of HIF-1α, recognizes ubiquitin-marked HIF-1α and drives its degradation under normoxia [17]. On the other hand, MDM2 is an E3 ligase of HIF-1α that forms a complex with p53 and leads to HIF-1α degradation in a p53-dependent manner under hypoxia [18]. Ubiquitination is a dynamic process that requires a series of enzymes (E1, E2, and E3) for conjugating ubiquitin to the target, and deubiquitinases (DUBs) remove the ubiquitin and maintain homeostasis [15]. USP20 is a DUB that counteracts VHL-mediated ubiquitination of HIF-1α [19]. USP8 and UCHL1 have been reported to remove ubiquitin from HIF-1α and HIF-2α, marked by VHL, to inhibit degradation [20]. MCPIP1 deubiquitylates HIF-1α to rescue its degradation [21]. These studies have reported the impact of a single E3 ligase or DUB on HIF-1α. In our study, we found that TARBP2 downregulates E3 ligases globally to attenuate the ubiquitination of HIF-1α, thereby resulting in enhanced expression of HIF-1α. These findings suggest that TARBP2 acts as a major modulator of HIF-1α ubiquitination status through controlling the expression of HIF-1α-related E3 ligases. Featured as an RNA-binding protein, TARBP2 is known to be a microRNA biogenesis factor involved in the cytoplasmic maturation of miRNAs [22,23,24]. Lin et al. found that the expression of TARBP2 is positively correlated with the stage in breast cancer, indicating that TARBP2 is an unfavorable prognostic marker [9]. The oncogenic effect of TARBP2 has also been reported in metastatic breast cancer indicating that the overexpression of TARBP2 destabilizes amyloid precursor proteins (APPs) and ZNF395 transcripts containing TARBP2-binding structural elements (TBSEs), thereby leading to the degradation of metastasis-suppressor transcripts associated with Alzheimer’s and Huntington’s disease, respectively [7]. On the other hand, TARBP2 is reported to be transcriptionally repressed in a PHD-dependent manner under hypoxia [25]. Let-7f-5p has also been found to be induced under hypoxia to suppress TARBP2 [26]. These studies indicate another layer of negative regulation from hypoxia to TARBP2. In our findings, we suggest a novel function of TARBP2 in regulating HIF-1α protein turnover, which transcriptionally downregulates HIF-1α-related E3 ligases. In TARBP2^high^ tumors, HIF-1α expression was found to be higher than that in TARBP2^low^ tumors, thereby suggesting that TARBP2 may serve as a crucial protein regulator of HIF-1α and exhibit an oncogenic role in cancer progression. 

## Data Availability

All images and raw data available on request. All other data are available from the corresponding authors.

## Availability of Data and Materials

Requests for materials should be address to Ming-Yang Wang (Department of Surgery, National Taiwan University Hospital, Taipei, Taiwan, no.7,8, Zhongshan S. RD., Zhongzheng Dist., Taipei City 10002, Taiwan. Phone: 886-2-2312-3456 (ext. 65083); email: suryang1971@hotmail.com).

## Abbreviations

TARBP2	TAR (HIV-1) RNA binding protein 2
HIF-1α	Hypoxia-inducible factor-1α
PHDs	Prolyl-4-hydroxylases
ODDD	Oxygen-dependent degradation domain
VHL	Von Hippel–Lindau protein
DUBs	Deubiquitinating enzymes
miRNA	MicroRNA
APP	Amyloid precursor protein
DMEM	Dulbecco’s modified Eagle medium
FBS	Fetal bovine serum
cDNA	Complementary DNA
IHC	Immunohistochemistry
TCGA	The Cancer Genome Atlas
CHX	Cycloheximide
CQ	Chloroquine
TBSEs	TARBP2-binding structural elements
SEM	Standard error of mean
KM plot	Kaplan–Meier plot
